# Circulating Tumor Cells Enumerated by a Centrifugal Microfluidic Device as a Predictive Marker for Monitoring Ovarian Cancer Treatment: A Pilot Study

**DOI:** 10.3390/diagnostics10040249

**Published:** 2020-04-23

**Authors:** Hyera Kim, Minji Lim, Jin Young Kim, So-Jin Shin, Yoon-Kyoung Cho, Chi Heum Cho

**Affiliations:** 1 Division of Hematology/Oncology, Department of Internal Medicine, Keimyung University Dongsan Hospital, Daegu 42601, Korea; hyerakim9465@gmail.com (H.K.); takgu@dsmc.or.kr (J.Y.K.); 2 Center for Soft and Living Matter, Institute for Basic Science (IBS), Ulsan 44919, Korea; ming4279@gmail.com; 3 Department of Biomedical Engineering, School of Life Sciences, Ulsan National Institute of Science and Technology (UNIST), Ulsan 44919, Korea; 4 Department of Obstetrics and Gynecology, Keimyung University Dongsan Hospital, Daegu 42601, Korea; hope2014@dsmc.or.kr

**Keywords:** circulating tumor cells, fluid-assisted separation technology, centrifugal microfluidic device, CA125, ovarian cancer

## Abstract

We investigated the size-based isolation and enumeration of circulating tumor cells (CTCs) using a centrifugal microfluidic device equipped with a fluid-assisted separation technology (FAST) disc. We further assessed the correlations among CTCs, cancer antigen-125 (CA125) levels, and clinical course of the disease in a prospective analysis of 47 serial blood samples collected at multiple time-points from 13 ovarian cancer patients. CTCs were isolated from whole blood using the FAST disc and were classified as epithelial cell adhesion molecule (EpCAM)/cytokeratin+, CD45−, and 4′,6-diamidino-2-phenylindole (DAPI)+. Mean CTC count at baseline was 20.2; 84.62% of patients had more than one CTC at baseline and had decreased CTCs counts after surgery and chemotherapy. The CTC counts in eight patients with complete responses were <3. CTC counts were correlated with CA125 levels in three patients without recurrence; they were elevated in three patients with recurrence and normal CA125 concentrations. CTC counts and CA125 levels showed high concordance with directional changes (increasing 71.4%; non-increasing 75.0%). CTC counts showed higher associations with clinical status, sensitivity (100.0% vs. 60.0%), positive predictive value (55.6% vs. 42.9%), and negative predictive value (100.0% vs. 87.5%) than CA125 levels. CTC counts were better associated with treatment response and recurrence than CA125 levels.

## 1. Introduction

Ovarian cancer is the eighth leading cause of death due to malignancies in Korean women and has a relatively poor prognosis compared to other gynecologic cancers [1]. Most patients are diagnosed with late-stage disease owing to the lack of symptoms and a useful screening tool. Cancer antigen-125 (CA125) is the biomarker mainly used for initial diagnosis and treatment monitoring in ovarian cancer [2]. CA125 concentrations >30 U/mL are generally considered to indicate recurrence in ovarian cancer. However, CA125 levels have poor sensitivity and specificity. Moss et al. [3] reported 88.6% sensitivity and 72.0% specificity of CA125 levels in 799 epithelial ovarian cancer patients. Moreover, non-malignant conditions such as endometriosis, pregnancy, and infection are well-known causes of elevated serum CA125 levels [4]. Therefore, CA125 levels are not useful for predicting prognosis and early recurrence after surgery and chemotherapy. Thus, identifying prognostic indicators reflecting current disease activity is crucial for patients with ovarian cancer.

Circulating tumor cells (CTCs) are shed into the bloodstream from primary tumors, recurrences, or metastases and possess both antigenic and genetic tumor-specific characteristics [5]. Previous studies have demonstrated the prognostic and predictive value of CTCs in patients with breast, colorectal, gastric, lung, and pancreatic cancers [6,7]. While the prognostic value of CTCs in ovarian cancer has also been investigated, the results were controversial [8]. Although similar immunocytochemistry methods were used for CTC quantification, Judson et al. reported no significant association between CTCs and overall survival (OS) and progression-free survival (PFS) [9]. Fan et al. reported a significant association between CTCs and PFS [10] while Pearl et al. reported significant relationships between CTCs and both OS and PFS [11]. Optimal strategies for CTCs should be established to predict disease activity, treatment response, or recurrence for the effective management of ovarian cancer.

Development of optimal methods for CTC enumeration in clinical settings remains a challenge because CTCs are rarely found in the peripheral blood, and the tumor status changes continuously with disease progression [12]. Currently, the CellSearch^®^ system (Menarini Silicon Biosystems, Inc., Huntingdon Valley, PA, US), which utilizes ferrofluids loaded with an epithelial cell adhesion molecule (EpCAM) antibody to capture CTCs [13], is the only system approved by the US Food and Drug Administration (FDA) system for clinical use in metastatic breast, prostate, and colorectal cancers [14]. However, its clinical utility in ovarian cancer has not yet been established. Poveda et al. reported a 14.4% positivity rate for a cut-off value of two CTCs/7.5 mL and observed no significant relationship between CTC counts and OS and PFS [15]. Behbakht et al. reported a 44.0% positivity rate for a cut-off value of 1 CTC/7.5 mL and observed no significant association between CTC counts and PFS [16].

This pilot study of 13 patients with ovarian cancer aimed to investigate a strategy for the enumeration and detection of CTCs based on a newly-developed centrifugal microfluidic device equipped with a fluid-assisted separation technology (FAST) disc and to demonstrate the correlations among CTC counts from the new device, CA125 concentrations, and clinical course of the disease.

## 2. Materials and Methods

### 2.1. Patients and Study Design

We prospectively recruited 13 women between December 2016 and August 2018. The patients were pathologically confirmed to have primary ovarian cancer and were scheduled to undergo a staging debulking surgery and to receive perioperative chemotherapy. They were treated at Keimyung University Dongsan Hospital and followed up until the date of death or the last visit. Written informed consent was obtained from all patients before enrollment. We collected 47 serial blood samples from the 13 patients at various time-points, including at diagnosis, before and after surgery or chemotherapy, and at radiological or clinical evaluation. A staging workup was performed before recruitment according to the 2014 International Federation of Gynecology and Obstetrics (FIGO) staging system. Information on age at diagnosis, type of treatment including surgery and chemotherapy, date of treatment and follow-up for evaluation, date of recurrence, initial FIGO stage, histology, response after surgery, and chemotherapy based on Response Evaluation Criteria in Solid Tumours (RECIST version 1.1), and serum CA125 levels was obtained from patient medical records. The study was approved by the Institutional Review Board of Keimyung University Dongsan Hospital (IRB 2016-03-014-001) and was conducted according to the principles of the Declaration of Helsinki.

### 2.2. FAST Disc Enumeration of CTCs

We used a commercial version of the FAST disc, the CD-PRIME^TM^ (Clinomics, Ulsan, Korea) comprised of two parts: a CD-CTC^TM^ Duo (disc) and a CD-OPR-1000^TM^ (disc operating machine), to isolate CTCs from the whole blood of ovarian cancer patients. The CD-CTC^TM^ Duo is a label-free, size-selective CTC isolation device that is used with the table-top-sized, stand-alone CD-OPR-1000^TM^ spinning system. The FAST and tangential flow filtration (TFF)-enabled CD-CTC^TM^ Duo allowed the rapid (>3 mL of whole blood/min) and clog-free isolation of CTCs from whole blood without any pre-treatment steps.

Immunostaining was conducted to identify the isolated cells on the membrane in the filtration chamber of the FAST disc. Isolated cells were fixed with 4% formaldehyde for 20 min at room temperature and permeabilized with 0.1% Triton-X 100 for 5 min. Subsequently, washing was followed by blocking with 10 µg/mL immunoglobulin G (IgG) in phosphate-buffered saline. The cells were stained with fluorescence-conjugated antibodies commonly used in CTC studies [17,18]: anti-cytokeratin (CK) (CAM5.2; BD, Franklin Lakes, NJ, USA mixed with AE1/AE3; eBioscience, San Diego, CA, USA) and anti-EpCAM (9C4; BioLegend, San Diego, CA, USA) for CTCs and anti-CD45 (H130; Life Technologies, Carlsbad, CA, USA) for white blood cells (WBCs); 4,6-diamidino-2-phenylindole (DAPI) was used for nuclear staining. Merged images of three different colors were used to distinguish tumor cells from WBCs. Cells that showed CK+ or EpCAM+ (FITC channel), CD45- (PE channel), and DAPI+ (DAPI channel) and were morphologically intact were identified as CTCs while cells with high CD45 expression were identified as WBCs. The fluorescence images of isolated cells on the membrane were automatically scanned on a Bioview workstation (BioView, Rehovot, Israel).

### 2.3. Cell Culture and Spike Experiment

SKOV3 and OVCAR3 ovarian cancer cell lines were purchased from the American Type Culture Collection (Manassas, VA, USA) and cultured with Roswell Park Memorial Institute (RPMI) medium including fetal bovine serum (FBS) (5% FBS for SKOV3 and 10% FBS for OVCAR3) and 1% antibiotics/antimycotics at 37 °C under 5% CO_2_. For the spiking experiments, the cells were labeled with CellTracker CMFDA (Life Technologies, Carlsbad, CA, USA) fluorescent dye before the experiment according to the manufacturer’s protocol.

### 2.4. Statistical Analysis

Exploratory statistical analyses [19] were performed to assess the clinical correlation between CTC counts and CA125 levels according to the treatment response. The treatment response was classified as complete response (CR) or progressive disease (PD) by a computerized tomography (CT) scan. Biomarker changes were also divided into increasing or non-increasing. Sensitivity, specificity, positive predictive value (PPV), and negative predictive value (NPV) were calculated to assess the concordance between the directional changes in CTC counts/CA125 levels and the treatment response. Fisher’s exact tests were used to determine the correlation between each marker and the treatment response. *p* < 0.05 was considered statistically significant. All statistical analyses were performed using IBM SPSS Statistics for Windows, version 25.0 (IBM Corp., Armonk, NY, USA).

## 3. Results

### 3.1. High-Throughput, Efficient, Label-Free Isolation of CTCs from Whole Blood Using the FAST Disc

**Figure 1:**
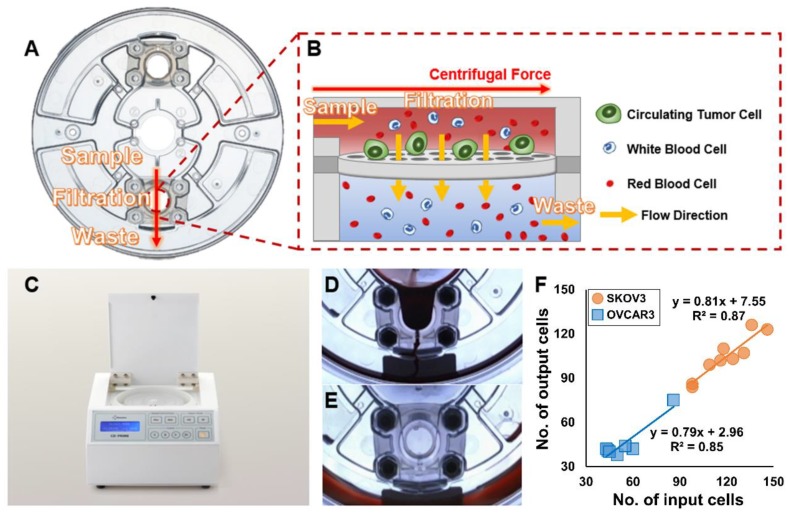
Mechanism and workflow of the CD-PRIME^TM^ (**A**) image of the CD-CTC^TM^ Duo; (**B**) schematic illustration of CTC isolation in the filtration chamber; (**C**) image of the CD-OPR-1000^TM^; (**D***,***E**) separate images of disc operation before and after washing; (**F**) FAST disc capture efficiency test with whole blood spiked with SKOV3 and OVCAR3 cells. The corresponding capture efficiencies were 87.5 ± 4.2% and 84.7 ± 9.1%, respectively (mean ± SD), respectively.

The FAST disc is a centrifugal microfluidic device with a track-etched polycarbonate membrane (pore size: 8 µm) for label-free CTC isolation. The FAST disc comprised three chambers: a sample loading chamber, a filtration chamber with a membrane, and a waste collection chamber (Figure 1A). In the filtration chamber, the membrane is assembled in a direction parallel to the centrifugal force. Thus, filtration through the pore occurs in a direction perpendicular to the centrifugal force, similar to that in TFF, which minimizes clogging. Furthermore, the design allows a uniform pressure drop across the whole membrane into the bottom chamber (underneath the membrane) filled with liquid (Figure 1B), which enables the isolation of intact cells with minimal pressure. A CD-OPR-1000^TM^ was used to operate the FAST discs. The experimental protocol was very simple. The FAST disc was loaded into the CD-OPR-1000^TM^ and centrifuged (Figure 1C). Both blood filtration (Figure 1D) and washing (Figure 1E) steps were conducted using this simple operation. By using the CD-PRIME^TM^, 3 mL of whole blood was processed within 1 min. Before applying the blood samples to the FAST disc, the performance of the FAST disc was confirmed using whole blood spiked with 40–150 SKOV3 and OVCAR3 cells. The mean capture efficiency was 87.5 ± 4.2% based on nine experiments with SKOV3 and 84.7 ± 9.1% based on seven experiments with OVCAR3 (Figure 1F).

### 3.2. Patient Characteristics

This study enrolled a total of 13 women with ovarian cancer. Their characteristics are shown in Table 1. The median age at diagnosis was 56 years (range 40–75 years). The histology of ovarian cancer was high-grade, serous carcinoma in nine patients. The initial FIGO stages in four, seven, and two patients were I–II, III, and IV, respectively. All patients underwent surgery and chemotherapy, and 11 patients achieved CRs. Six patients had a recurrence in the lung or liver or peritoneal seeding. The baseline CA125 concentrations were high in most patients, except for those with stage I disease; CTCs were detected in the baseline samples of 84.62% of patients (11/13). All patients underwent debulking surgery including total abdominal hysterectomy, bilateral salpingo-oophorectomy, bilateral pelvic lymph node dissection, para-aortic nodal dissection, appendectomy, and omentectomy. Thirteen patients received platinum doublet-based adjuvant chemotherapy, such as paclitaxel plus carboplatin with or without bevacizumab for 4–6 cycles, and four patients received neoadjuvant chemotherapy before surgery due to an unresectable status. After therapy, samples from 10 patients were collected and tested. In 90% of patients, CA125 levels and CTC counts were lower than those at baseline. Only one patient (Patient 6) had increased CTC counts after therapy. This atypical patient also had a normal CA125 concentration (<30 U/mL) at baseline.

**Figure 2:**
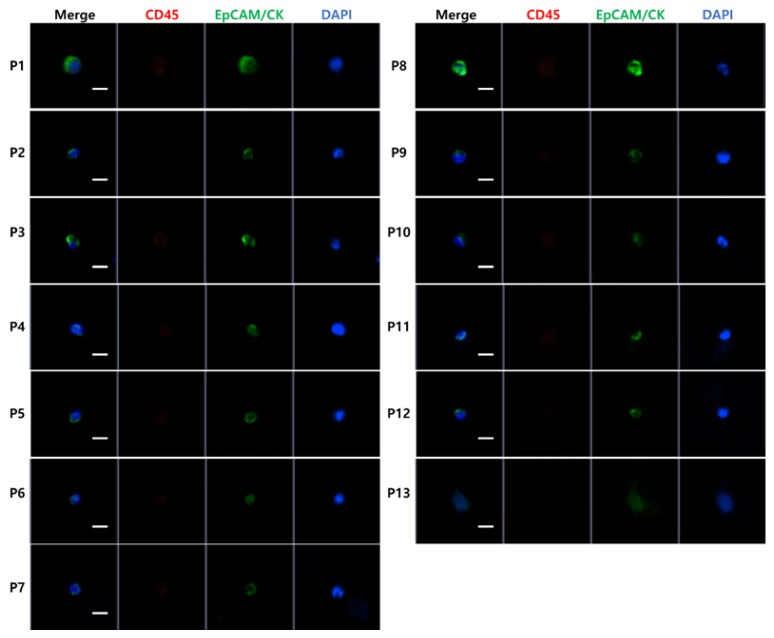
Examples of fluorescence images of patient-driven CTCs. CTCs are defined as DAPI+, EpCAM/CK+, and CD45- cells. White line: scale bar = 10 µm.

From each patient sample, isolated cells were stained and discriminated based on the conventional criteria for CTC identification: EpCAM/CK+, DAPI+, and CD45–. Figure 2 shows representative CTC images from patient samples.

### 3.3. Detection of CTCs and Their Correlation with CA125 Concentrations

At baseline, 84.62% of patients were positive for CTCs, with one or more CTCs detected in 3 mL of blood (range 1–76). The mean and median CTC counts in all patients at baseline were 20.2 and 6.0, respectively. The median follow-up duration was 22.7 months (range 5.2–28.7). Most patients presented with decreased CTC counts after surgery and chemotherapy, except for two patients with no CTCs at baseline. At the time of CR, the CTC counts in eight patients were <3.

**Figure 3:**
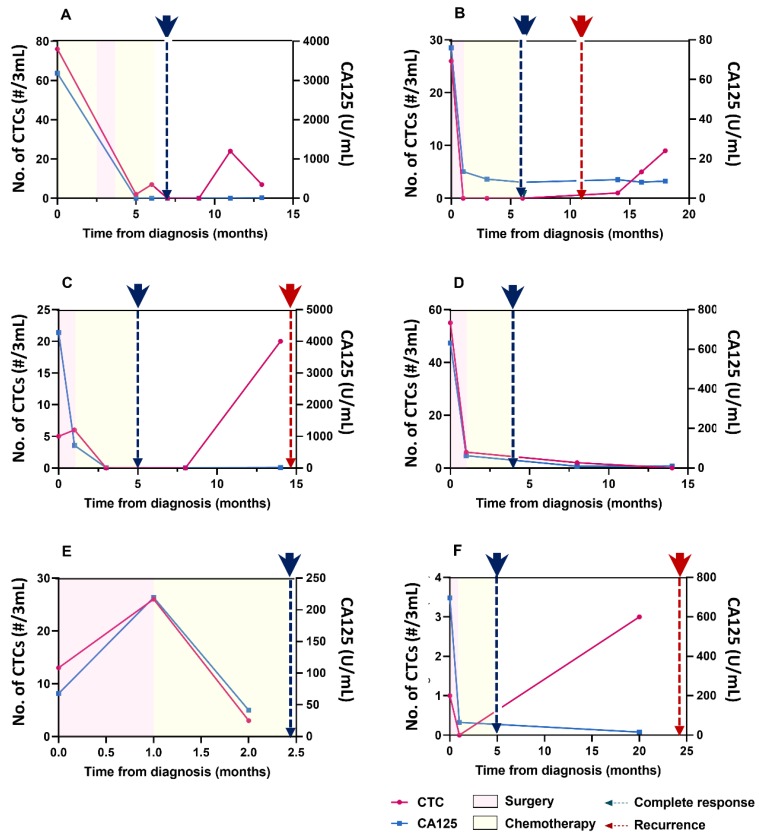
Correlations among circulating tumor cell (CTC) counts, cancer antigen-125 (CA125) concentrations, and clinical course in six patients (**A**) Patient 1: after perioperative chemotherapy and surgery, the CTC count and CA125 level decreased to almost the baseline levels, and a complete response was observed on computed tomography (CT) at the end of chemotherapy; (**B**) Patient 2: at the end of chemotherapy and surgery, the CTC count and CA125 concentration were 0/3 mL and 8.1 U/mL, respectively, and a complete response was noted. Five months later, an increase in the CTC count was followed by recurrence, although the CA125 concentration remained within the normal range; (**C**) Patient 3: following surgery and chemotherapy, the CTC count and CA125 concentration were 0/3 mL and 11.3 U/mL, respectively, and a complete response was noted. Five months later, an increase in the CTC count was noted with normal CA125 concentrations, followed by a recurrence of peritoneal seeding; (**D**) Patient 5: following surgery and chemotherapy, a durable complete response was observed on CT, with a CTC count and CA125 concentration of 2/3 mL and 9.3 U/mL, respectively, which further decreased to 0/3 mL and 10.0 U/mL, respectively; (**E**) Patient 8; the patterns of CTC counts and CA125 concentrations were similar throughout treatment; (**F**) Patient 9: 20 months after diagnosis, the CTC counts increased slightly, but the CA125 concentration remained in the normal range, and recurrence was detected in the peritoneum.

The correlation analysis between CTC counts and CA125 levels according to the clinical course of the disease included patients (1) with >3 blood samples, (2) whose initial CA125 levels were higher than normal (30 U/mL), and (3) who showed CR based on imaging findings after surgery. Based on these criteria, six patients were selected and were divided into the no recurrence and recurrence groups. The three patients in the no recurrence group showed similar patterns for CTC counts and CA125 levels, which were correlated with the clinical course. Patient 1 was diagnosed with ovarian cancer, high-grade serous carcinoma, and had an initial FIGO stage of IIIC. The pre-treatment CTC count and CA125 concentration were 76/3 mL and 3186.5 U/mL, respectively. After perioperative chemotherapy and surgery, both parameters decreased to baseline levels, and CR was observed on computed tomography (CT) performed at the end of chemotherapy (Figure 3A). Patient 5 was diagnosed with ovarian cancer, high-grade serous carcinoma, and had an initial FIGO stage of IIIA2. The pre-treatment CTC count and CA125 concentration were 55/3 mL and 631.9 U/mL, respectively. After surgery and chemotherapy, a durable CR was observed on CT; the CTC count and CA125 concentration were 2/3 mL and 9.3 U/mL, respectively, which further decreased to 0/3 mL and 10.0 U/mL, respectively, after 14 months (Figure 3D). Patient 8 was diagnosed with ovarian cancer, clear cell carcinoma, and had an initial FIGO stage of IIB. The pre-treatment CTC count and CA125 concentration were 13/3 mL and 67.8 U/mL, respectively. The changes of both parameters were similar during treatment, and a CR was observed after two months (Figure 3E).

The three patients in the recurrence group showed similar CTC and CA125 patterns. However, the CTC counts increased before or after recurrence while the CA125 concentrations remained in the normal range. For example, Patient 2 was diagnosed with ovarian cancer, high-grade serous carcinoma, and had an initial FIGO stage of IIIC. The pre-treatment CTC count and CA125 concentration were 26/3 mL and 76.0 U/mL, respectively. After chemotherapy and surgery, the corresponding values were 0/3 mL and 8.1 U/mL, respectively, and a CR was observed on CT. Recurrence was detected five months later with the appearance of new lung nodules. An increase in the CTC count was followed by a recurrence, although the CA125 concentration remained within the normal range (Figure 3B). Patient 3 was diagnosed with ovarian cancer, high-grade serous carcinoma, and had an initial FIGO stage of IIIC. The pre-treatment CTC count and CA125 concentration were 5/3 mL and 4278.5 U/mL, respectively. After surgery and chemotherapy, these values decreased to 0/3 mL and 11.3 U/mL, respectively, and a CR was observed on CT. Five months later, an increase was observed in the CTC count, but the CA125 level remained in the normal range; this was followed by a recurrence of peritoneal seeding (Figure 3C). Patient 9 was diagnosed with ovarian cancer, high-grade serous carcinoma, and had an initial FIGO stage of IIB. Twenty months after diagnosis, the CTC count increased slightly, but the CA125 concentration remained in the normal range; a recurrence was detected in the peritoneum (Figure 3F).

**Figure 4:**
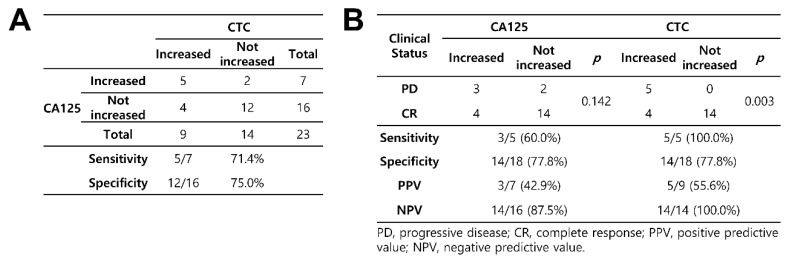
Clinical correlation between CTC counts and CA125 levels according to the treatment response. (**A**) concordance between the changes in CA125 levels and CTC counts for increasing (sensitivity) and non-increasing (specificity) values; (**B**) concordance between treatment response and the changes in CTC counts/CA125 levels for increasing (sensitivity) and non-increasing (specificity) values.

All changes in CTC counts and CA125 levels were analyzed to check for concordance and association with the corresponding clinical assessments. First, to confirm the concordance between the CA125 concentration and CTC count, each point of change in the six cases was classified into one of the four categories: both increased, only CTC increase, only CA125 increase, and no change in either (no change or decrease). The concordance in cases in which both the values increased was 71.4%. However, the concordance in cases in which there were no increases in either value was 75.0%. According to the directional concordance analysis, the CTC count was highly correlated with a change in CA125 concentrations (Figure 4A). To analyze the association with the corresponding clinical status, each point of change in the six cases was classified according to recurrence. Both CA125 levels and CTC counts had the same specificity of 77.8%. However, CTC counts showed a higher sensitivity (100.0% vs. 60.0%), PPV (55.6% vs. 42.9%), and NPV (100.0% vs. 87.5%) than CA125 concentrations. CTC counts were significantly associated with clinical status (*p* = 0.003) (Figure 4B).

## 4. Discussion

This pilot study successfully confirmed the performance of the FAST disc for CTC enumeration in ovarian cancer patients. Our assessment of the clinical course over a long period demonstrated that CTC counts showed a higher association with treatment response than CA125 levels. We observed a >70% concordance between the changes in CTC counts and CA125 levels, and the change in CTC counts showed higher sensitivity, PPV, and NPV than the change in CA125 levels. Pearl et al. monitored the treatment response of six patients with >6 CTCs and with CA125 measurements and reported that changes in CTC counts but not CA125 levels antedated changes in clinical response, from progression to CR during and after chemotherapy and relapse [11]; this finding is consistent with those of the present study.

The increasing number of elderly patients with ovarian cancer, which is a result of improved life expectancy, underscores the need for optimal treatment guidelines for this population. Previously, most elderly patients received supportive care without cancer treatment or single-modality treatments. Because of their poor performance status and comorbidities, it is challenging to apply standard therapy to elderly patients. However, the results of recent studies suggest that elderly patients could also benefit from surgery and chemotherapy, with decreased perioperative mortality. Neoadjuvant chemotherapy and less invasive cytoreductive surgery are the preferred strategies in elderly ovarian cancer patients [20,21]. Improved management of elderly patients with ovarian cancer requires careful selection of eligible patients and the identification of useful tumor biomarkers for early diagnosis and treatment.

The importance of tumor biomarkers for improving survival in gynecological cancer patients has been increasingly emphasized. Previous studies have tried to identify new systematic methods with high sensitivity and specificity in gynecological cancer [22,23]. Diverse methods have been used to develop tumor biomarkers for ovarian cancer: abnormal DNA methylation or *BRCA1*/*BRCA2* gene mutations through gene chip technology [24,25], serum proteomics [26], autoantibodies sequence with immunohistochemistry [27], B7-H4 (one of the T cell costimulatory molecule B7 family) [28], and lysophosphatidic acid [29]. In addition, some markers were shown to detect pre-invasive (p16ink4a, p16, E-cadherin, Ki67, pRb, p53) and invasive (CEA, SCC-Ag, CD44) lesions in cervical cancer [23]. The present study sought to assess CTCs as a novel biomarker in ovarian cancer following the isolation of CTCs with an improved device.

We developed a centrifugal microfluidic device equipped with a FAST disc. This device is based on label-free, size-based CTC isolation without the need for sample pre-treatment and employs TFF. This system enables clog-free, ultrafast (>3 mL/min), viable CTC enrichment with gentle pressure drops (~1 kPa). This device was tested in 142 patients with seven different types of cancers, including breast, lung, and stomach cancers [30]. The sensitivity and specificity of CTCs in gastric cancer (85.3% and 90.3%, respectively, for a cut-off of two CTCs/7.5 mL in 116 patients) [31], esophageal squamous cell carcinoma (85.3% and 90.0%, respectively, for a cut-off of two CTCs/7.5 mL in 116 patients) [32], and colorectal cancer (75.0% and 100.0%, respectively, for a cut-off of five CTCs/7.5 mL in 74 patients) [33] have been demonstrated. Moreover, Baek et al. found that patients with colorectal cancer with ≥5 CTCs showed a trend toward poor OS and PFS and frequent vascular invasion (*p* = 0.035) [33]. In addition, several studies comparing the FAST disc to other commercially available platforms, such as the immunoaffinity-based CellSearch systems [34] and the AccuCyte^®^-CyteFinder^®^ (Rarecyte) selection-free system [35] have demonstrated the advantages of the FAST disc system, including its high-throughput, label-free isolation of CTCs and high detection rate. Furthermore, this FAST disc system used a label-free method; thus, captured CTCs can be retrieved relatively easily for downstream analysis. This enables single-cell and genetic analysis for targeted therapy. Based on the results of previous studies on other cancer types, we evaluated the clinical validation of this disc to enumerate CTCs in ovarian cancer patients. The results of the present pilot study provide insightful evidence for future studies with larger cohorts of patients with ovarian cancer.

CTCs are considered a liquid biopsy and a non-invasive method for diagnosis and prognosis. Samples can be easily collected at multiple time-points throughout treatment, which allows real-time monitoring of treatment response and drug resistance for all metastatic cancers [36]. Moreover, CTC analysis enables the detection of multiple mutations within a single cell, which contributes to an improved understanding of tumor heterogeneity and clonal evolution to establish a potential connection between mutational status and pathway activation by combining CTC genomics and transcriptomics [37]. However, the limitations include the lack of large-scale studies with standardized approaches to enrich and detect CTCs [38]. Most of the studies on ovarian CTCs using the FDA-approved CellSearch system reported negative results due to its comparatively low accuracy in CTC detection and the limited availability of patients with ovarian cancer. In a multicenter, randomized, phase III study including 216 ovarian cancer patients, only 14.4% were found to have two or more CTCs before treatment using this system [15]. Liu et al. reported that CTC counts were not significantly correlated with survival outcomes in 78 patients with ovarian cancer using this system (newly-diagnosed patients, median time to progression [TTP] 14.0 vs. 16.5 months, *p* = 0.88; recurrent disease, median TTP 3.8 vs. 6.4 months, *p* = 0.13) [39].

Previous studies have sought to enhance CTC yield and purity based on biophysical properties such as size, deformability, or dielectric susceptibility. By using reverse transcription-quantitative polymerase chain reaction (RT-qPCR) to detect CTCs based on their overexpression of the cyclophilin C gene (PPIC), Obermayr et al. showed a significantly higher presence of CTCs in the platinum-resistant patient group than in the platinum-sensitive group, which was also related to poor prognosis in the follow-up of 93 patients with ovarian cancer [40]. Pear et al. reported that serial measurements using CAM+ selection of CTCs could predict therapeutic responsiveness in 31 patients with ovarian cancer who received standard paclitaxel plus carboplatin therapy [11]. Kim et al. found that the positive detection of postoperative CTCs using the tapered-slit filter platform was associated with inferior PFS rates in 39 patients with stage III/IV ovarian cancer (18.8% vs. 57.1%, *p* = 0.077) [41]. We used size-based isolation first to achieve high throughput, label-free, high-yield CTCs isolation and then used the gold standard enumeration by immunostaining with EpCAM/CK+ to define CTCs.

This study has several limitations. First, the small sample size was enrolled from a single center. Second, the numbers and timing of blood sampling were irregular; therefore, it was difficult to compare samples from different patients to make statistically significant comparisons during the time course of therapy. Third, we only quantified EpCAM/CK positive CTCs, although the enumeration method was not EpCAM marker-sensitive. Fourth, we only measured the number of CTCs per sample and did not compare our findings to those of other enumeration methods or analyze other molecular characteristics of the CTCs. Therefore, further studies with larger sample sizes and more in-depth characterization are required.

In conclusion, we used a FAST disc to successfully isolate sufficient CTCs from the blood of ovarian cancer patients. We demonstrated that CTC counts were better associated with treatment response and recurrence throughout the clinical course than CA125 concentrations. To our knowledge, this is the first report on the evaluation of CTCs in ovarian cancer patients using a newly-developed centrifugal microfluidic device equipped with a FAST disc based on label-free isolation. The results implied the potential utility of CTCs as a predictive marker for monitoring treatment response in patients with ovarian cancer. Further large-scale prospective studies are needed to validate the FAST disc as a diagnostic tool for personalized management of ovarian cancer.

## Figures and Tables

**Table 1:** Clinical characteristics of patients with ovarian cancer.

No.	Age at Diagnosis (years)	Initial FIGO Stage	Histology	Initial CA125 (U/mL)	Initial CTCs (/3 mL)	Treatment Response	CA125 after Therapy (U/mL)	CTCs after Therapy (/3 mL)	No. of Blood Samples
1	65	IIIC	High-grade serous carcinoma	3186.5	76	CR	5.8	0	7
2	67	IIIC	High-grade serous carcinoma	76	26	CR	8.1	0	7
3	55	IIIC	High-grade serous carcinoma	4278.5	5	CR	11.3	0	5
4	53	IC	Mucinous carcinoma	18.2	64	CR	8.5	1	5
5	49	IIIA2	High-grade serous carcinoma	631.9	55	CR	9.3	2	4
6	56	IC	High-grade serous carcinoma	25.7	0	CR	7.7	3	4
7	75	IV	Adenocarcinoma with serous carcinoma	10,000	0	PR	244.5	0	4
8	40	IIB	Clear cell carcinoma	34.6	13	CR	41.6	3	3
9	59	IIB	High-grade serous carcinoma	696.4	1	CR	65.1	0	3
10	46	IIIC	Clear cell carcinoma	2432.8	6	CR	153.1	0	2
11	47	IV	High-grade serous carcinoma	553.3	2	CR	NA	NA	1
12	66	IIIC	High-grade serous carcinoma	3399.8	10	CR	NA	NA	1
13	65	IIIC	High-grade serous carcinoma	8767.4	4	PR	NA	NA	1

FIGO, International Federation of Gynecology and Obstetrics; CTCs, Circulating Tumor Cells; CR, complete response; PR, partial response.
